# Supplementary material for: COBRA: cell-type-specific orthogonal batch effect removal algorithm in single cell RNA-sequencing data
Source: Bioinformatics. 2026 Sep 2;42(9):btag660. doi: 10.1093/bioinformatics/btag660 (PMC13612174; doi:10.1093/bioinformatics/btag660)

Supplementary Figure S1. UMAP visualization for murine atlas dataset

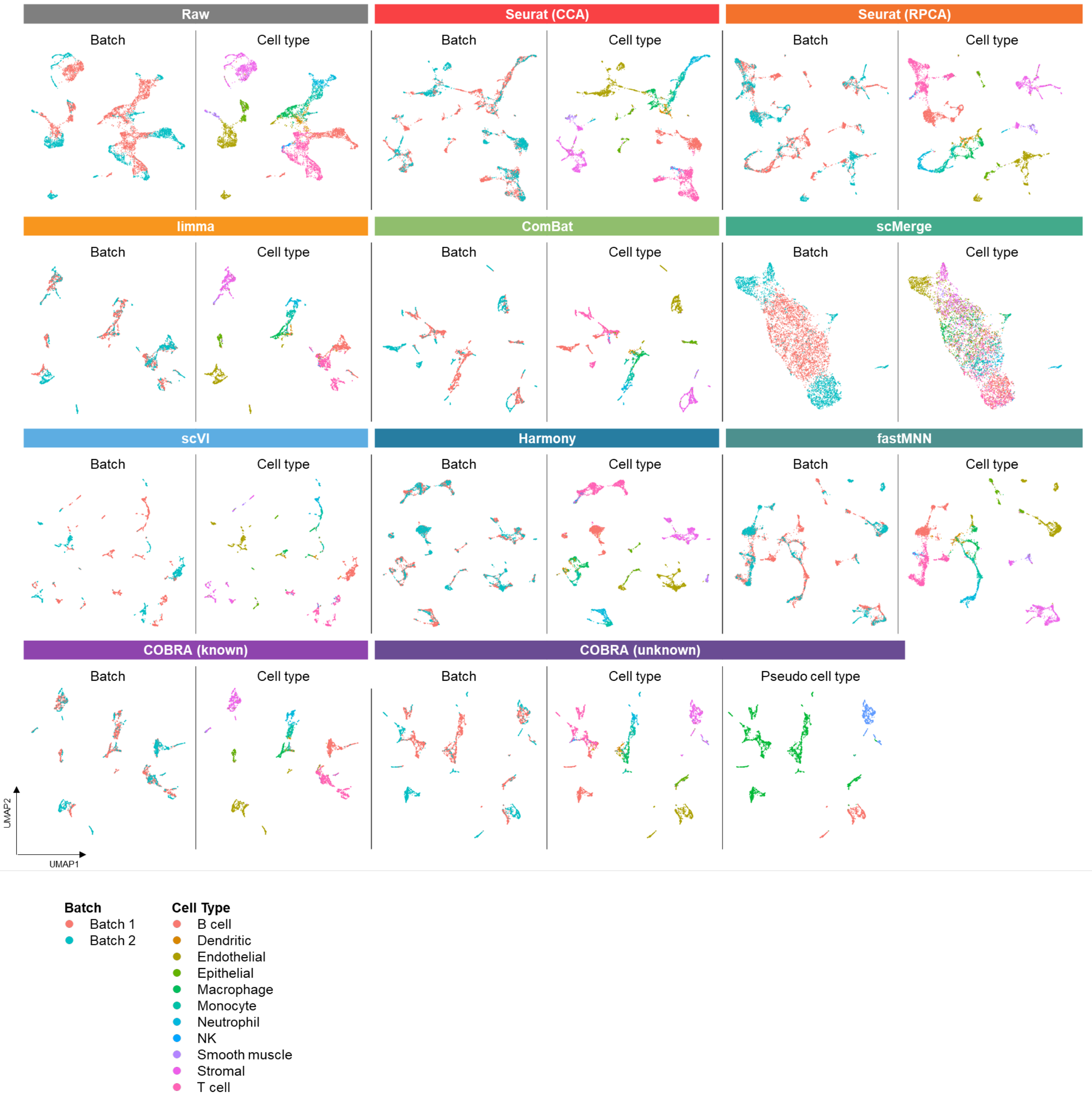

Supplementary Figure S2. UMAP visualization for human pancreas dataset

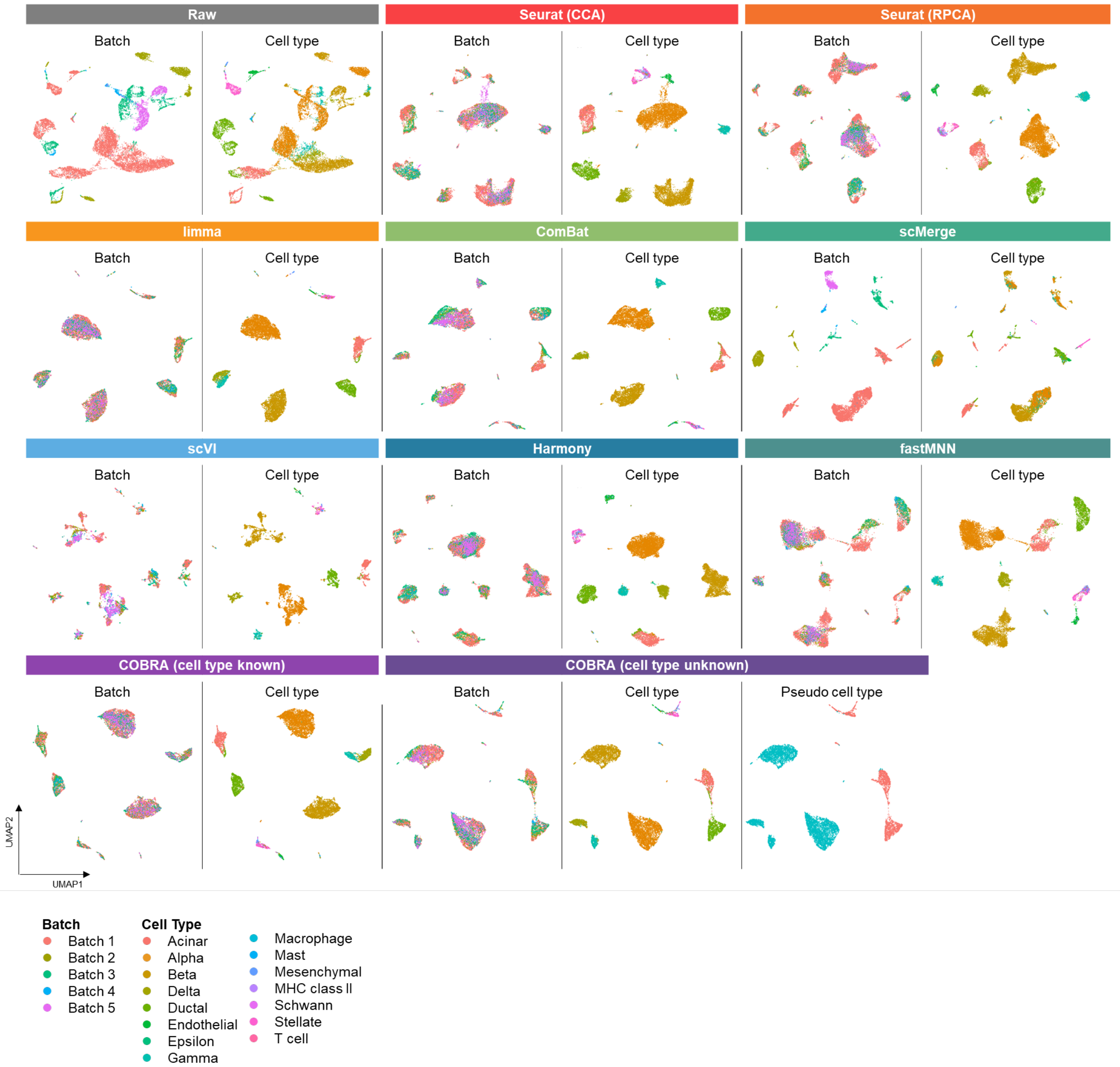

Supplementary Figure S3. UMAP visualization for human PBMC dataset

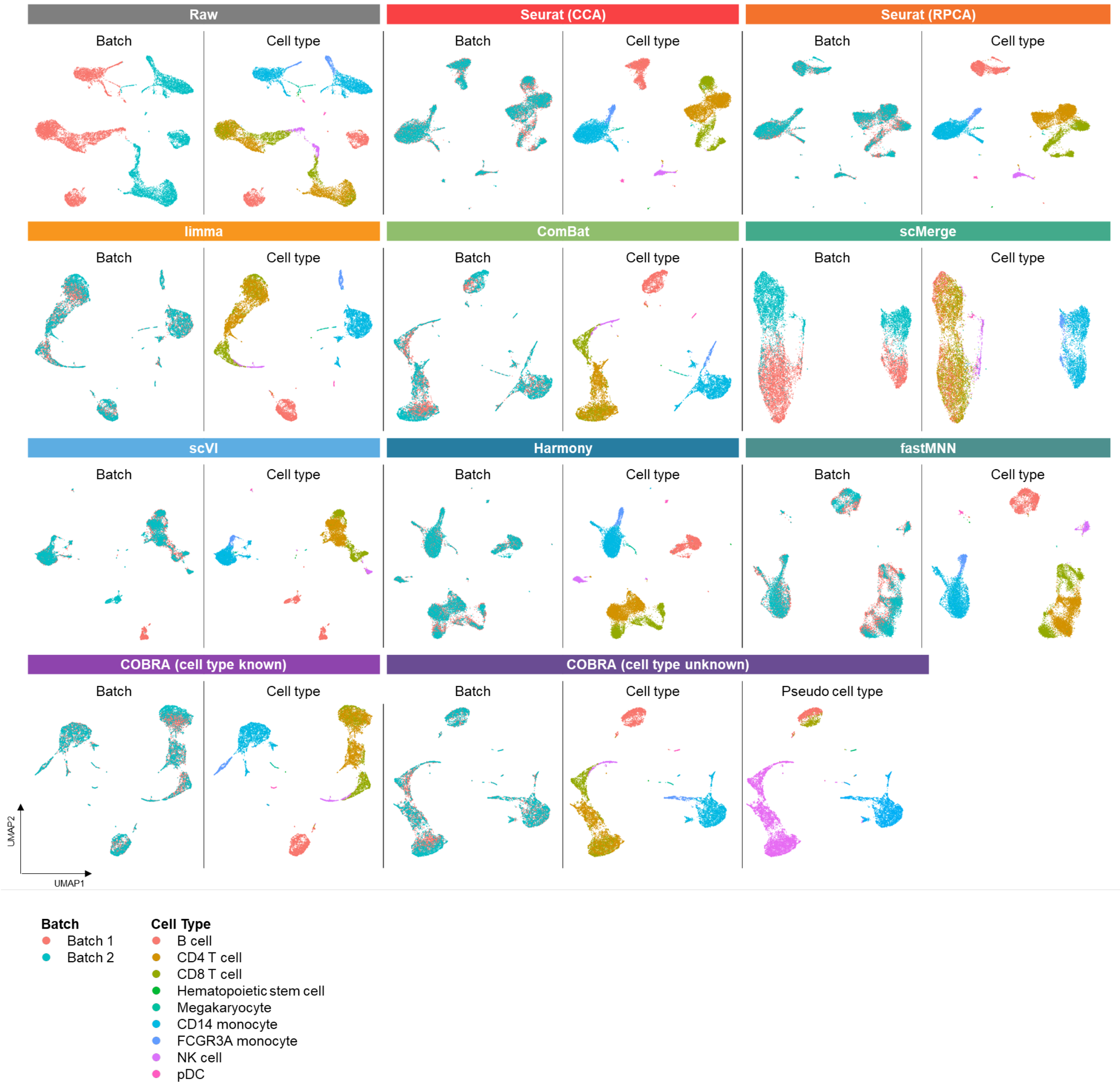

Supplementary Figure S4. UMAP visualization for mouse retina

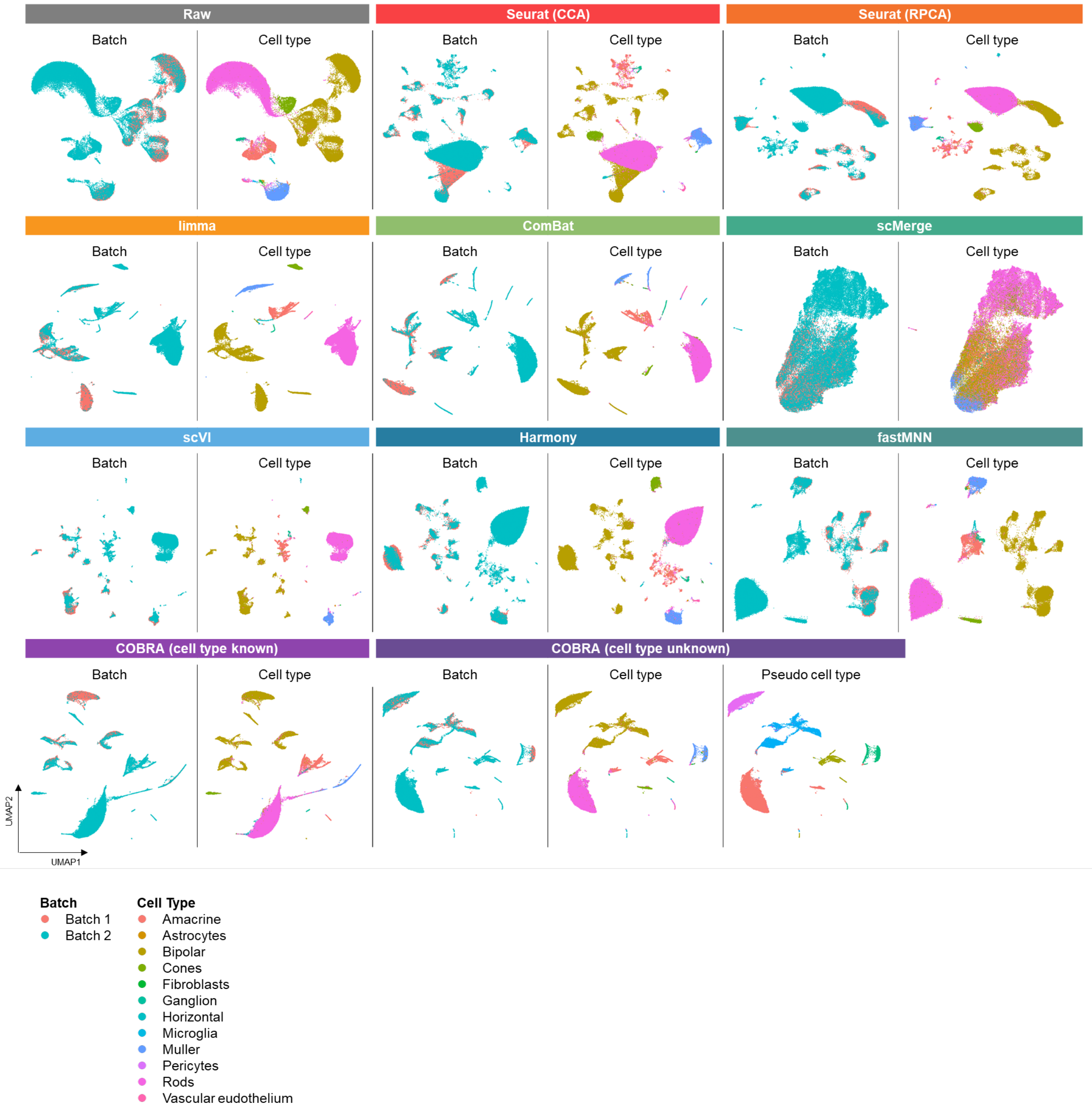

Supplementary Figure S5. UMAP visualization for mouse hematopoietic stem and progenitor cells dataset

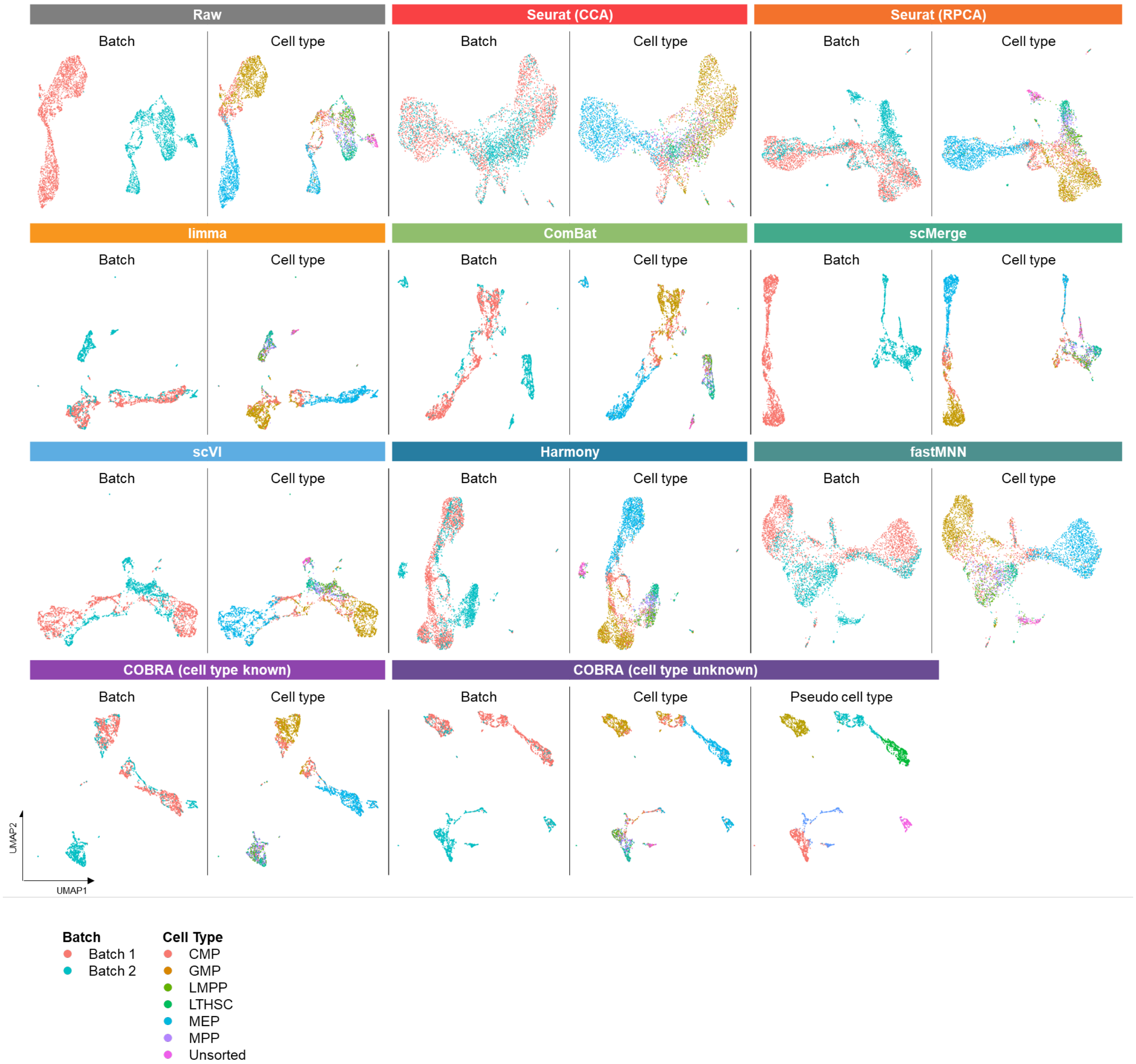

Supplementary Figure S6. UMAP visualization for mouse brain dataset

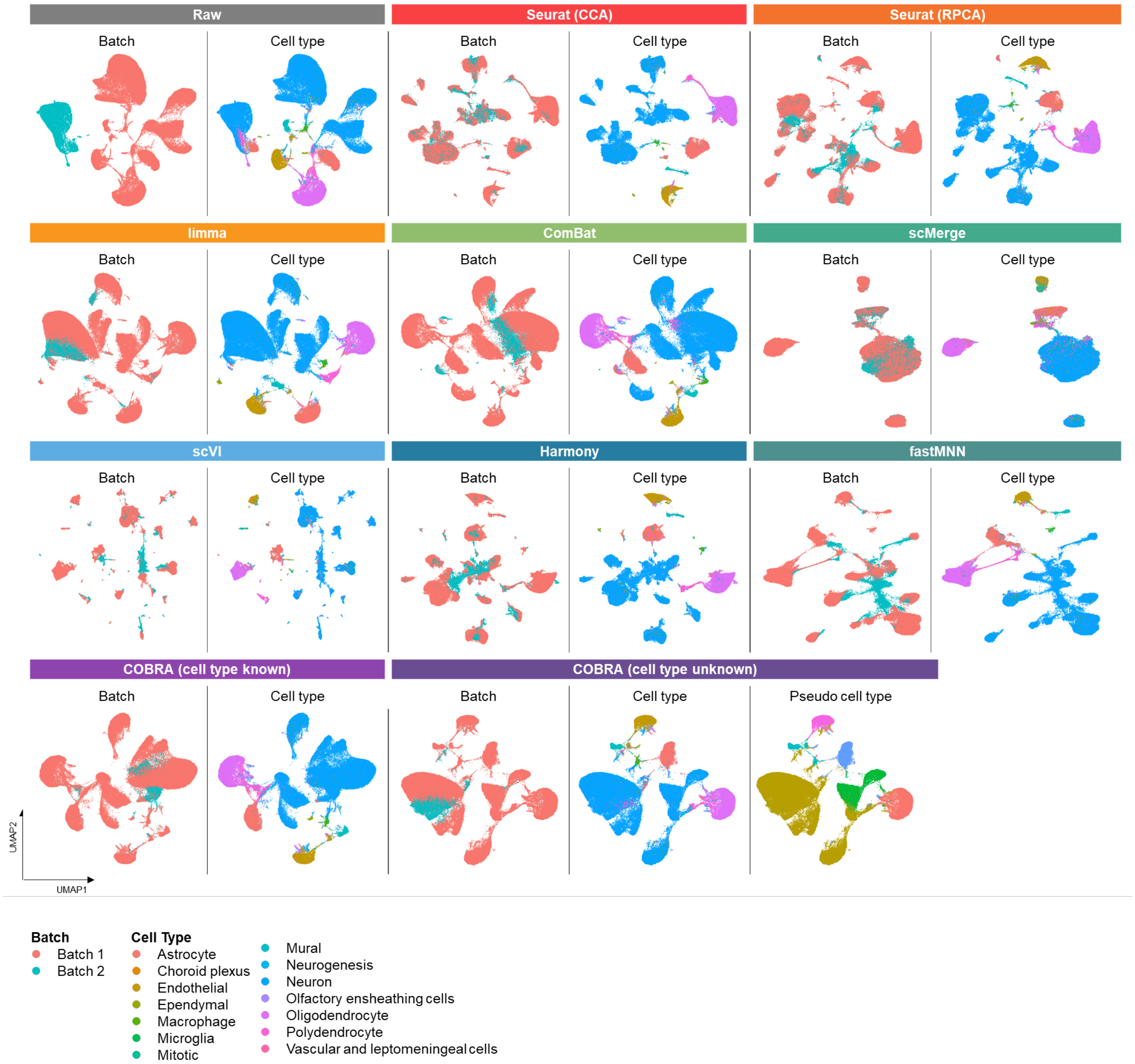

Supplementary Figure S7. UMAP visualization of batch integration for (a) simulation 1 dataset and (b) cell line dataset

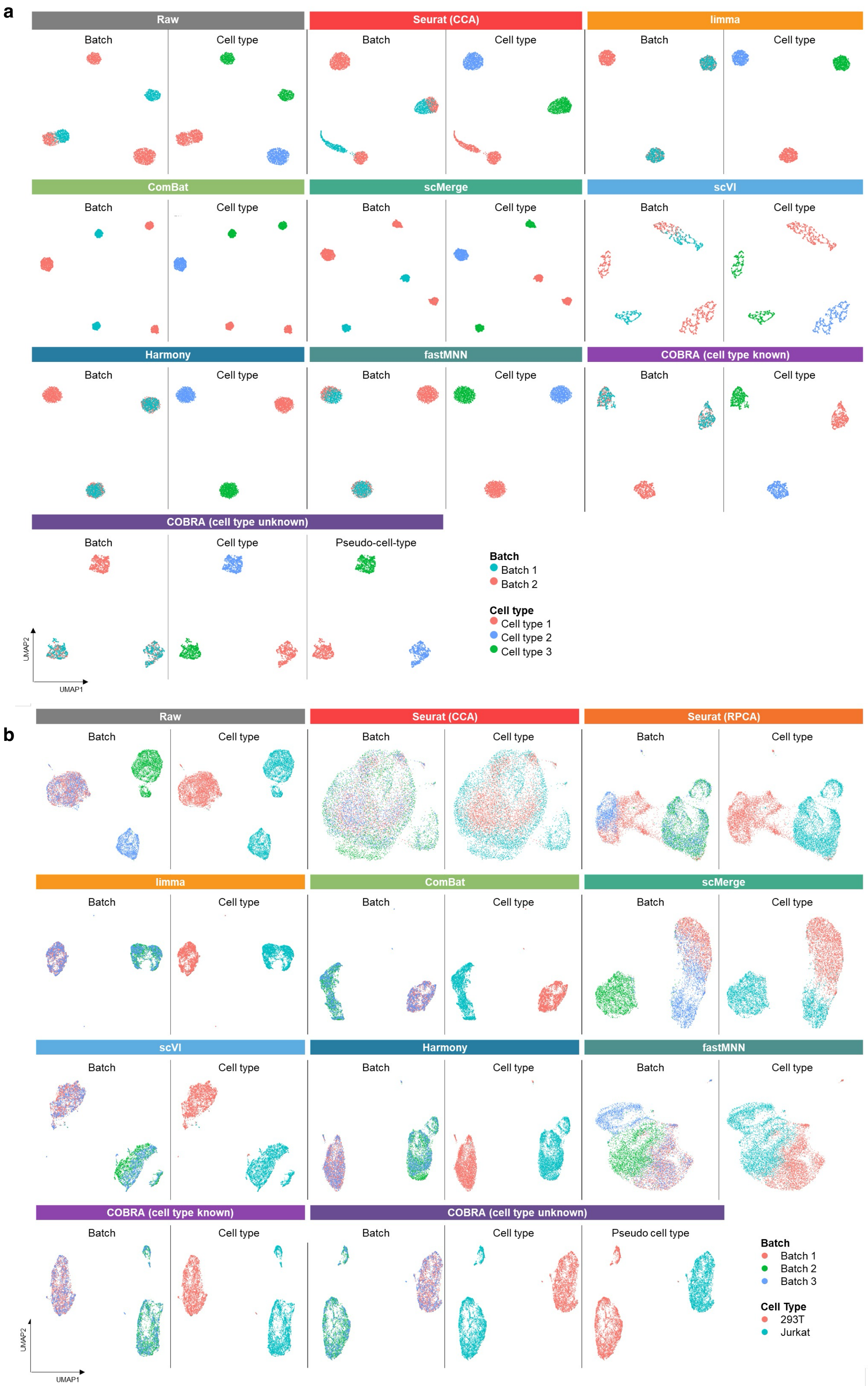

Supplementary Figure S8. UMAP plot of batch integration for simulation 2 (a) raw dataset and (b) COBRA integrated dataset by batch (left) and cell type (right)

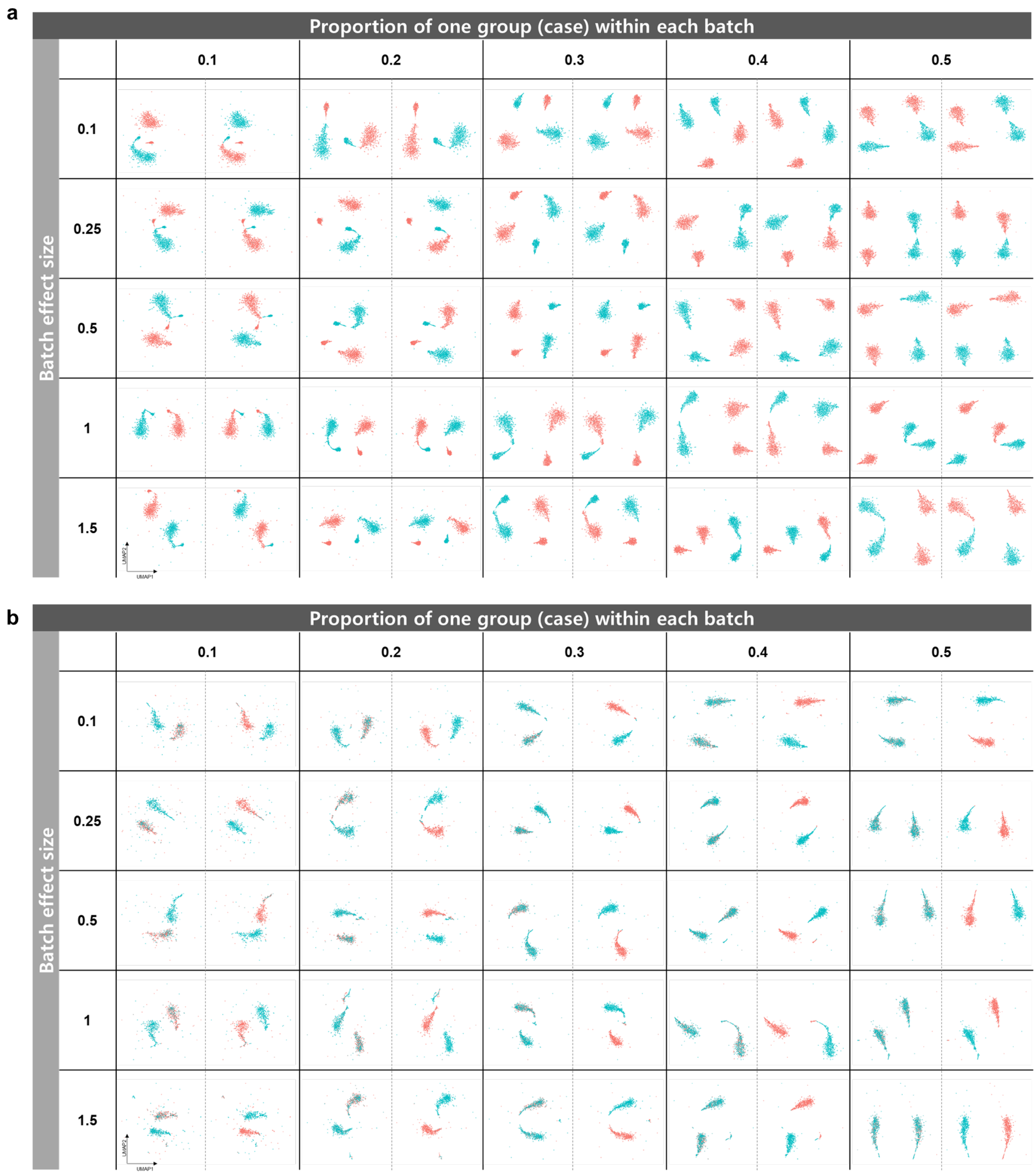

Supplementary Figure S9. UMAP visualization for T2D dataset

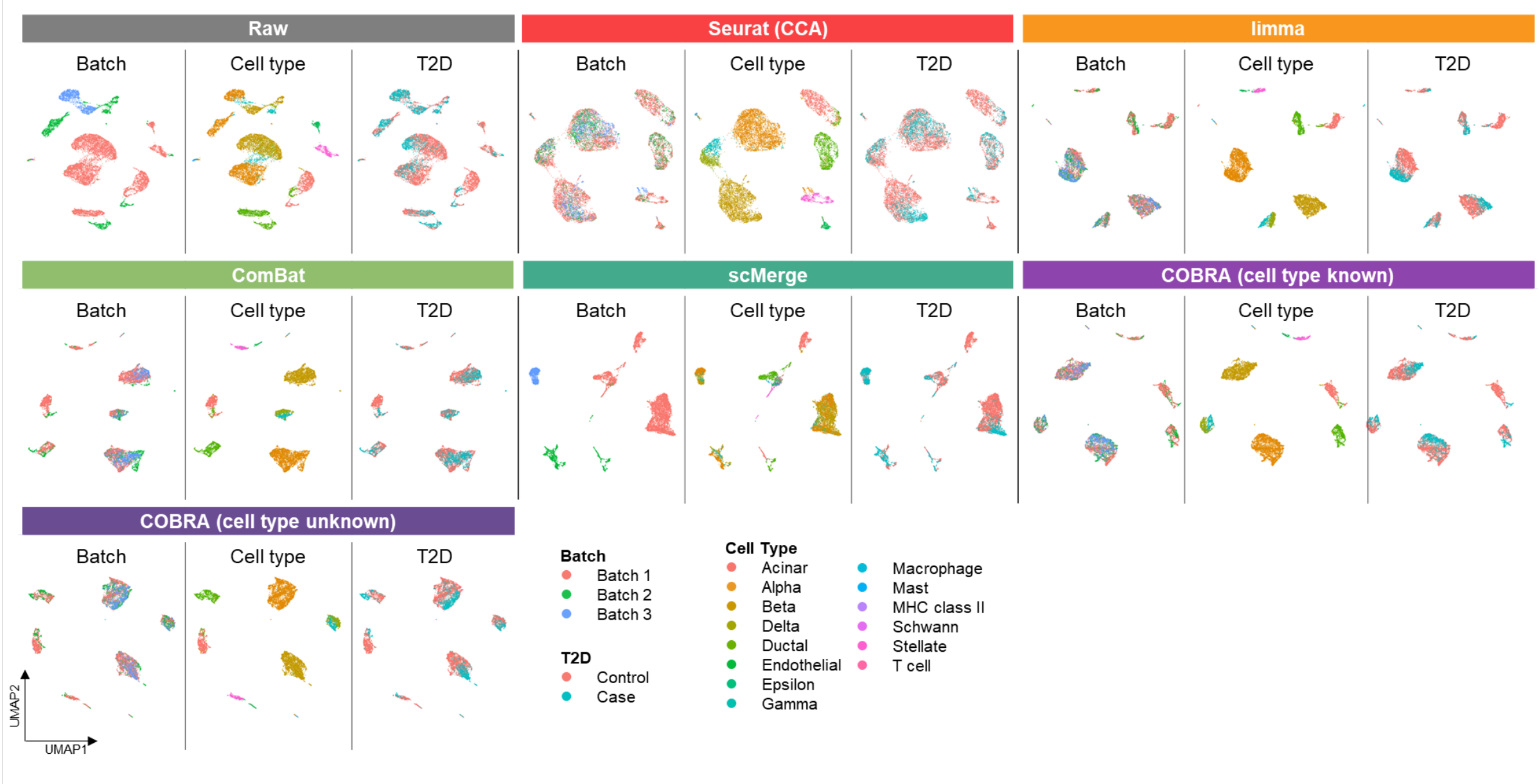

Supplementary Figure S10. UMAP visualization for COVID-19 dataset

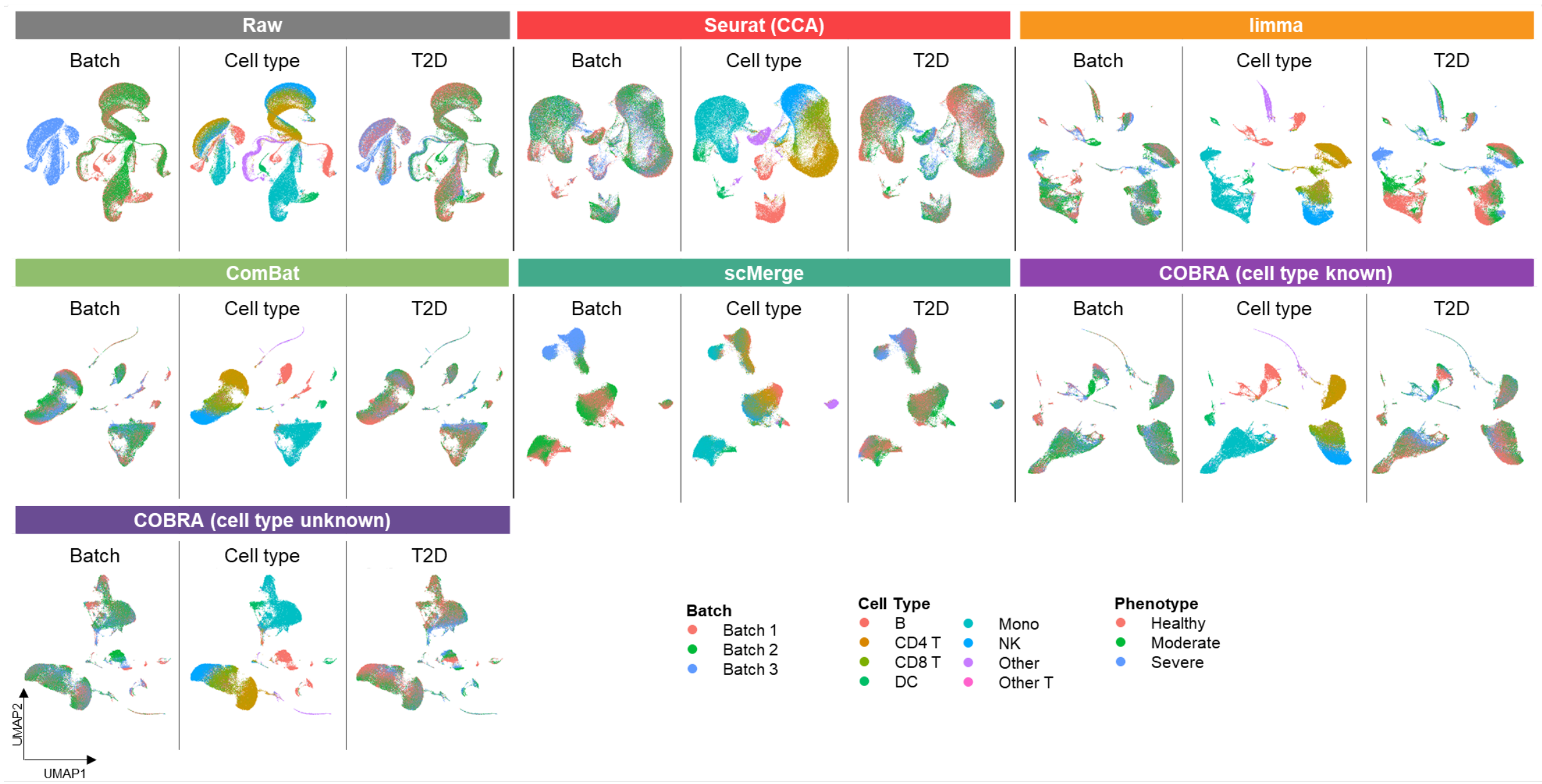

Supplementary Figure S11. Computational scalability of COBRA as a function of the number of interaction terms.

Runtime and peak memory usage were benchmarked for COBRA, limma, and ComBat across 16 configurations defined by the number of batches (2, 4, 6, 8) and cell types (2, 4, 6, 8), with a fixed dataset size of 10,000 cells and 2,000 genes.
(a) Runtime in seconds. (b) Peak memory usage in MiB.

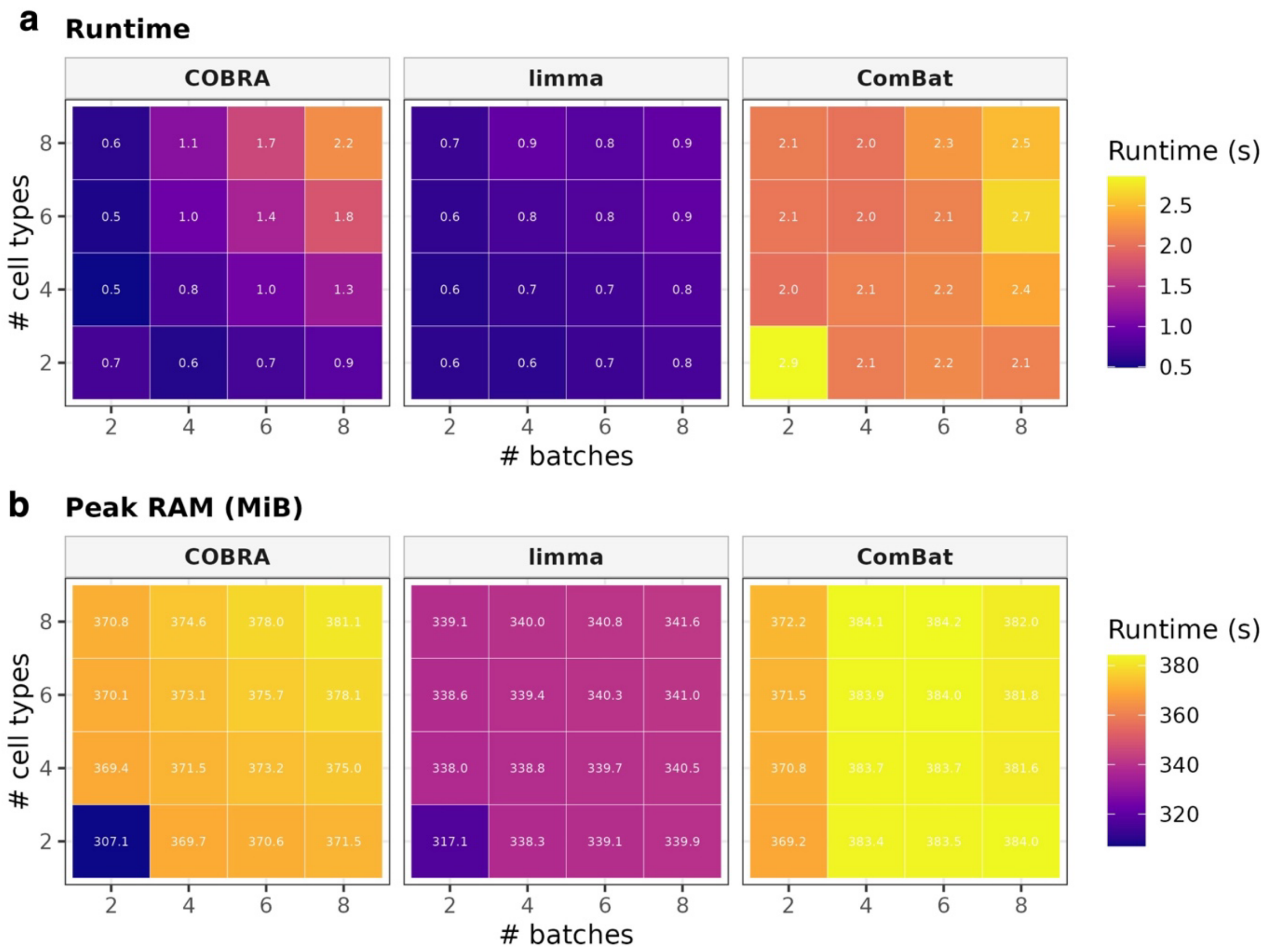

Supplementary Figure S12. Robustness of pseudo-cell type estimation.

Sensitivity of COBRA's iterative pseudo-cell type estimation was assessed across 100 independent simulated datasets, each containing 3,000 cells, 2,000 genes, 3 batches, 6 true cell types, and 2 phenotypic groups.

(A) Selected K across 100 simulations using 20 PCs. Each point represents the final K from one simulation.

(B) Adjusted Rand index (ARI) between pseudo-cell labels and true cell-type labels across 100 simulations. Points are colored by the selected K.

(C) Selected K as a function of the number of PCs (10 to 50), with 20 random seeds per setting.

(D) ARI between pseudo-cell labels and true cell types as a function of the number of PCs.

(E) Sensitivity of downstream batch correction quality to the choice of K. For each simulation, COBRA was run with K fixed at values from 6 to 10 (spanning K* ± 2 around the automatically selected K*).

(F) Comparison of K-means with Louvain clustering.

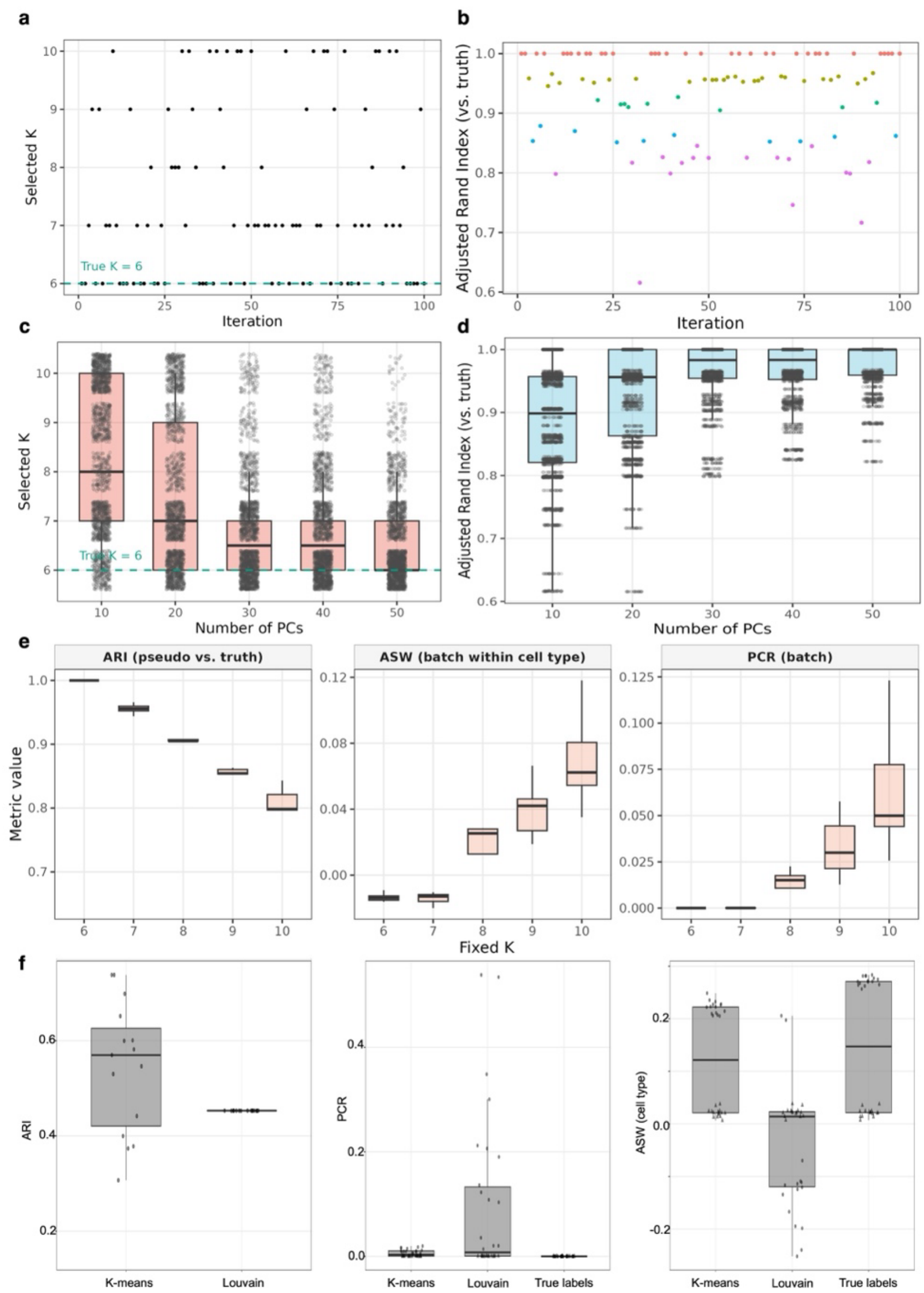

Supplementary Figure S13. COBRA preserves continuous trajectory structure while removing batch effects in a Splatter-simulated differentiation process.

Synthetic data generated using Splatter with splatSimulate in "paths" mode. The simulation comprised 2,000 genes and 3,000 cells divided equally between two batches (1,200 cells each). Three lineages were specified with a bifurcating (Y-shaped) topology, in which paths 2 and 3 branched from path 1 (path.from = c(0, 1, 1)). Each path was resolved over 100 steps (path.nSteps), and lineage proportions were set to 0.35/0.30/0.35 (group.prob). The probability of differential expression across paths was set to 0.30 (de.prob). Batch effects were introduced through Splatter's multiplicative batch factors, with location and scale parameters both set to 0.10 (batch.facLoc = batch.facScale = 0.10).

(a) UMAP visualizations before correction **(b)** UMAP visualization after correction with COBRA **(c)** lineage-resolved scatterplots of inferred slingshot pseudotime against the ground-truth step, shown separately for each lineage.

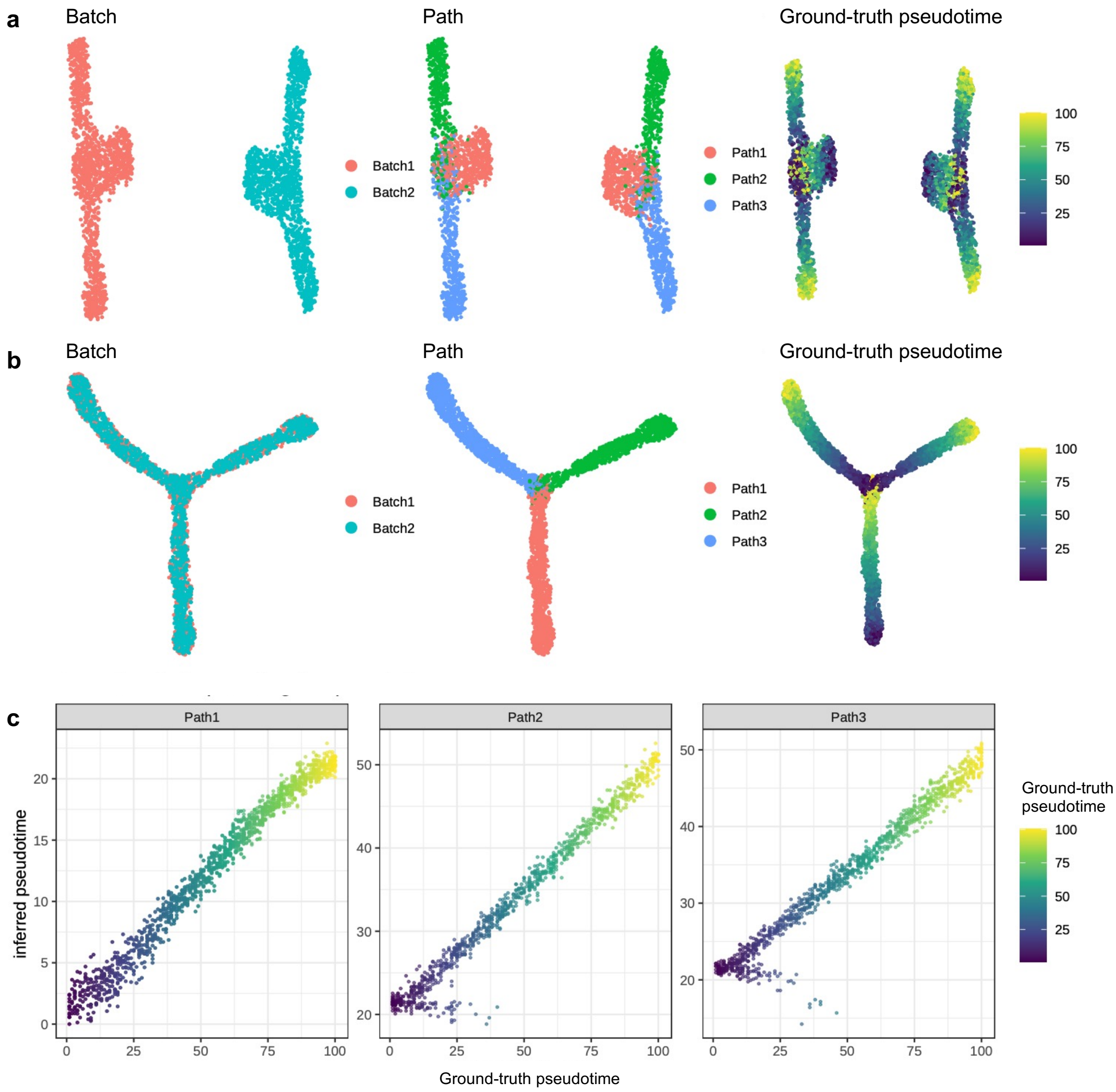

Supplement: Supplementary file 1 [file btag660_supplementary_data.pdf]
